# Effect of Processing Temperature and the Content of Carbon Nanotubes on the Properties of Nanocomposites Based on Polyphenylene Sulfide

**DOI:** 10.3390/polym13213816

**Published:** 2021-11-04

**Authors:** Kamil Dydek, Paulina Latko-Durałek, Agata Sulowska, Michał Kubiś, Szymon Demski, Paulina Kozera, Bogna Sztorch, Anna Boczkowska

**Affiliations:** 1 Faculty of Materials Science and Engineering, Warsaw University of Technology, 141 Wołoska, 02-507 Warsaw, Poland; paulina.latko@pw.edu.pl (P.L.-D.); agata.sulowska.stud@pw.edu.pl (A.S.); szymon.demski.dokt@pw.edu.pl (S.D.); paulina.kozera@pw.edu.pl (P.K.); anna.boczkowska@pw.edu.pl (A.B.); 2 Faculty of Power and Aeronautical Engineering, Warsaw University of Technology, 24 Nowowiejska, 00-665 Warsaw, Poland; michal.kubis@pw.edu.pl; 3 Centre for Advanced Technologies, Adam Mickiewicz University in Poznań, 10 Uniwersytetu Poznańskiego, 61-614 Poznań, Poland; bognasztorch@gmail.com

**Keywords:** PPS, carbon nanotubes, thermal analysis, electrical properties, rheological properties

## Abstract

The study aimed to investigate the effect of processing temperature and the content of multi-wall carbon nanotubes (MWCNTs) on the rheological, thermal, and electrical properties of polyphenylene sulfide (PPS)/MWCNT nanocomposites. It was observed that the increase in MWCNT content influenced the increase of the complex viscosity, storage modulus, and loss modulus. The microscopic observations showed that with an increase in the amount of MWCNTs, the areal ratio of their agglomerates decreases. Thermogravimetric analysis showed no effect of processing temperature and MWCNT content on thermal stability; however, an increase in stability was observed as compared to neat PPS. The differential scanning calorimetry was used to assess the influence of MWCNT addition on the crystallization phenomenon of PPS. The calorimetry showed that with increasing MWCNT content, the degree of crystallinity and crystallization temperature rises. Thermal diffusivity tests proved that with an increase in the processing temperature and the content of MWCNTs, the diffusivity also increases and declines at higher testing temperatures. The resistivity measurements showed that the conductivity of the PPS/MWCNT nanocomposite increases with the increase in MWCNT content. The processing temperature did not affect resistivity.

## 1. Introduction

Modern technological developments have created a need for new materials or the expansion of parameters and functional features of currently available products. In recent years, high-performance thermoplastics, also known as engineering polymers, have been the subject of study for many scientists. This was a result of the exceptional properties of the polymers, their ease of forming, and many possibilities of modifications. Polymers containing aromatic units, such as polyether ether ketone (PEEK), polyphenylene sulfide (PPS), and poly(ether sulfone) (PES), are widely used in electrical [1], automotive [2], and chemical industries [3,4]. Polyphenylene sulfide (PPS) is one of the most used engineering semi-crystalline thermoplastic due to its properties and relatively high temperature resistance (melting point 275 °C) [5,6]. PPS has good chemical resistance to most organic solvents, including alcohols, ketones, chlorinated aliphatic compounds, esters, and liquid ammonia [7]. It is also resistant to atmospheric factors, such as moisture and UV radiation. It has a low coefficient of thermal expansion and is, therefore, a material with excellent dimensional stability. Its natural non-flammability is also an advantage in many applications [8,9]. Although neat PPS possess exceptional properties, in the case of many applications, they are insufficient; thus, a great deal of research focuses on improving them or adding new functional properties through the modification of PPS [10].

One of the most popular methods of PPS modification is the production of polymer blends [11]. J. Z. Liang [12] manufactured PPS/Polycarbonate (PC) blends to investigate the influence of PC content on heatproof properties. It has been demonstrated that when the PC weight fraction is less than 20%, the distortion temperature of the PPS/PC blend is lower than that of neat PPS. The reason is the lower heatproof property of PC than PPS and an insufficient number of physical nodes of the macromolecular chains between the PPS and PC. The number of nodes will increase with higher PC content, resulting in an improved heatproof property. Z. Ma et al. [13] fabricated microcellular foams from high-performance PPS/PEEK blends and compared their performance with solid PPS/PEEK blends. The conclusion was that PEEK could improve the impact toughness of the PPS blend due to their interaction, and the presence of PEEK greatly accelerates the crystallization process of PPS. Microcellular foaming improves the crystallinity, specific tensile strength, and impact strength of the PPS/PEEK blends and decreases the storage modulus and loss factor. Another method of PPS modification is the production of PPS composites with inorganic fillers, i.e., TiO_2_ and ZnO. This solution allows for a significant improvement in tribological properties compared to pure PPS—by adding 6 wt% of TiO_2_, the material wear index was reduced 100 times, so it can be successfully used in bearings, brake pads, and gears [14,15].

Although methods of improving parameters are available and a new application of PPS blends and composites is observed, there are still areas that require new modifications, e.g., in the field of conductive materials. The electrical performance of polymer matrix composites (PMCs) can be improved by adding metal or carbon particles, i.e., carbon nanotubes (CNTs). R. K. Goyal et al. [16] prepared PPS composites with copper (Cu) particles by first mixing and then injection molding to investigate the influence of metal particles on various properties of composites, including electrical properties. The results showed that the percolation threshold is around 6 vol% of Cu particles and the electrical conductivity increases to 18.5 vol% of Cu particles due to the infinite conductive cluster in the matrix. However, on further increasing the Cu content, the conductivity decreased due to the porosity of the composite. J. Yang et al. [17] obtained (PPS)/multi-wall carbon nanotube (MWCNT) nanocomposites by melt mixing PPS with raw MWCNTs without any pre-treatment, to investigate the mechanical and electrical properties of such composites. The melt blending of PPS with MWCNTs was conducted using a mixer at a temperature of 300 °C for 10 min. Considering electrical properties, it was observed that there is a gradual decrease of resistivity before MWCNTs is increased to 5 wt%. Then, a sharp decrease of the resistivity of almost five orders of magnitude (from 10^−12^ to 10^−7^ Ω·cm) was achieved, as the content was increased from 5 to 7 wt%. However, with a higher MWCNT content, only an insignificant decrease in resistivity was observed. M. Park et al. [18] investigated the enhancement of PPS composites’ interfacial, electrical, and flexural properties by introducing CFs coated with MWCNTs based on electrophoresis. PPS composites filled with seized CFs or with CFs coated with MWCNTs were fabricated using a twin-screw extruder. The interfacial shear strength of the PPS composites was improved by about 41.7% due to the MWCNTs introduced on the surface of CFs. Introducing MWCNTs on the CF surface improved the electrical conductivity of the composites by about 78%, probably because MWCNTs are excellent electrically conductive fillers. Additionally, the percolation threshold of the PPS composites filled with the MWCNT coated CFs based on electrophoresis was lower than that of PPS composites filled with resized CFs. Moreover, low thermal conductivity, which is characteristic of polymers, limits a range of applications in many cases where heat dissipation is required. This parameter can also be modified by using highly thermally conductive fillers. Y. Seki et al. [19] prepared PPS-based composites modified with a maximum of 5 wt% of synthetic graphite that showed an approximately triple increase in thermal diffusivity in the through-thickness direction and the 32-fold larger increase for the in-plane direction compared with neat PPS. S. Deng et al. [20] demonstrated an impact of graphene nanoplates on the thermal conductivity of PPS. Thermal conductivity for the neat matrix was reported as 0.219 W m^−1^·K^−1^. It increased up to 1.156 W m^−1^·K^−1^ in the case of 30 wt% content of graphene nanoplates. G. Junwei [21] presented even higher thermal conductivity for PPS/Graphene nanoplates composite. An almost 20-fold increase in thermal conductivity was achieved by using a similar weight fraction of filler.

Another example of processing of PPS composites is to use them in the form of fibers, which combine moderate thermal properties with excellent chemical resistance, but few studies have included the modification with a high volume of carbon nanotubes (CNT). Xing et al. [22] manufactured PPS fibers with functionalized graphene nanoplatelets (GNPs) via melt spinning to improve the oxidation resistance of the composite. The result shows that the addition of GNPs influences the surface roughness of fibers and increases the degree of crystallinity of PPS. Kulpinski et al. [6] manufactured PPS fibers and nonwovens by the melt-blown method modified with CNT and carbonyl iron microparticles at low content (up to 1 wt%). The mechanical properties of fibers with fillers were worse than neat PPS because of the chosen production method. The addition of CNTs improved the electrical properties of fibers, and manufactured nonwovens obtained electromagnetic properties. EMI shielding of nonwovens was tested and the results were satisfying. Moreover, PPS can be also used as a material for the filament to 3D printing [23]. Parans Paranthaman et al. used PPS/NbFeB composites to produce permanent magnets using the FDM technique [24].

The aim of this article is to optimize the processing temperature of PPS/MWCNT nanocomposites and the content of MWCNTs so that in the developed material, it would be possible to successfully produce nanocomposite fibers, which in the next stage will be used to produce conductive, thermoplastic nonwovens using the thermo-pressing method [25]. Thermoplastic nonwovens containing MWCNTs can be used as interlayers or surface finish to modify carbon fiber reinforced polymers (CFRPs) to improve electrical and mechanical properties [26,27,28]. Quan et al. [29] used PPS/MWCNT nonwovens produced wet-laid process, where the MWCNTs were applied using the airbrush technique, and then, the nonwovens were used to improve the properties of CFRP composites. In the case of improving the electrical properties, thermoplastic nonwovens with the addition of MWCNTs allow the creation of conductive paths between the layers of carbon fibers, which in turn reduces the CFRP resistivity [30]. Additionally, no reports were found in the literature about the impact of processing using a twin-screw extruder on the properties of PPS/MWCNT nanocomposites. To sum up, the use of PPS/MWCNT nanocomposites as a material for the production of thermoplastic nonwovens by thermo-pressing method is an innovative approach, so far unheard of in the literature, which justifies the need to select and optimize the processing temperature as well as the content of MWNCTs in PPS/MWCNT nanocomposites in order to verify their functional properties. 

## 2. Materials and Methods

### 2.1. Materials

Fortron^®^ PPS (Celanese, Pasadena, TX, USA) was used as a polymer matrix to produce PPS/MWCNT nanocomposites, whereas MWCNTs with the trade name NC7000 from Nanocyl (Sambreville, Belgium), synthesized through catalytic carbon vapor deposition process, were used as a conductive nanofiller. The average diameter of an MWCNT is 9.5 nm, its length is 1.5 µm, and its purity >95%. Additionally, Nanocyl manufactured and delivered masterbatch in the form of pellets, using a semi-industrial production line equipped with a twin-screw extruder. The masterbatch contained 10 wt% MWCNT. Then, using the laboratory twin-screw extruder HAAKE MiniLab (ThermoFisher Scientific, Waltham, MA, USA), mixing neat PPS with delivered masterbatch, nanocomposites PPS/MWCNT were produced with the following MWCNT concentrations: 2 wt%, 4 wt%, 6 wt%, and 8 wt%. Moreover, to check the impact of the processing conditions, three different temperatures were applied: 290, 305, and 320 °C at a constant rotational speed of the screws, which was 50 rpm. To investigate the effect of the processing temperature on the dispersion of MWCNTs in the polymer matrix, the masterbatch was also extruded at three different temperatures, namely 290, 305, and 320 °C.

The test specimens were produced using a laboratory HAAKE MiniJet Pro (Thermo Fisher Scientific) injection molding machine, where the mold temperature was 140 °C, and the injection and hold pressures were 800 and 700 bar, respectively. The temperatures in the cylinder, where the material was plasticized, were 290, 305, and 320 °C, corresponding to the processed nanocomposite material.

### 2.2. Measurement Methods

The rheological properties of the material were measured using an oscillatory ARES rheometer (Rheometric Scientific Inc., TA Instruments, New Castle, DE, USA) in parallel plate geometry mode. Firstly, the amplitude sweep test was performed, to choose the appropriate strain values from the linear elastic range. Afterward, using the selected strain (10%), a dynamic oscillatory stress-controlled rotational test was performed at 320 °C with a frequency sweep from 0.1 to 100 Hz. To examine how the addition of MWCNTs affects the rheological properties of PPS, the complex viscosity, storage modulus, and loss modulus were collected. Specimens for the rheological analysis were prepared directly from the pellets by injection molding into rounds having a diameter of 1.5 mm and a thickness of 2 mm.

The state of MWCNTs’ dispersion has been characterized by transmission optical microscopy. Samples were prepared using Ultramicrotome Leica EM UC6 (Leica Microsystems, Wetzlar, Germany) equipped with a chamber for low-temperature processing. The materials were cut with a diamond knife at the temperature −10 °C into slides with 2 μm thickness. Furthermore, samples were deposited on the surface of a microscopic glass and observed through a light transmission microscope PZO Biolar (Biolar, Warsaw, Poland). The optical micrographs were analyzed using the digital image processing software ImageJ (National Institutes of Health and the Laboratory for Optical and Computational Instrumentation, Madison, WI, USA). The MWCNTs’ agglomerate area ratio was defined as the ratio of the cumulative area of the MWCNTs’ agglomerate system relating to the micrograph area, and this ratio was presented in a percentage [31]. Only MWCNT agglomerates with diameters higher than 1 µm were used for analysis. To have sufficient statistics of measured values, at least seven optical illustrating micrographs were used for calculation.

The thermal stability of the PPS/MWCNT composites was investigated by thermogravimetric analysis (TGA) using a TGA Q500 (TA Instruments, New Castle, DE, USA). Samples with a weight of around 11 mg were placed in an aluminum crucible and heated from 0 to 820 °C in a nitrogen atmosphere with a heating rate of 10 °C/min and a flow rate of 10 mL/min and 90 mL/min. The degradation temperature at 2% (T2%) and 5% (T5%) weight loss, as well as the temperature of the maximum weight loss rate (Td), were determined from the obtained TGA curves.

Thermal properties of materials were studied using a Q1000 Differential Scanning Calorimeter (TA Instruments, New Castle, DE, USA). Samples with a weight of 8.0 + 0.2 mg were placed in an aluminum hermetic pan. Firstly, the samples were equilibrated at 0 °C, then heated to 340 °C with a scan rate of 10 °C/min, and cooled to 0 °C with a scan rate of 5 °C/min. Finally, they were heated again to 340 °C with a scan rate of 10 °C/min. The process was conducted in a nitrogen atmosphere. Using the Universal V4.5A TA software, the melting point (Tm) was determined from the second heating curve and the crystallization temperature (Tc) was determined based on the cooling curve. The crystallinity content (Xc) of the PPS composites was calculated from Equation (1):

(1) $$X_{c}\left(\%\right)=\frac{\Delta H_{c}}{\Delta H{{}^{\circ}}_{m\;}\left(1-x\right)}\Delta 100\%$$

where ΔH_c_ is the enthalpy of melting taken as the area under the melting peak from the second heating curve, ΔH°_m_ is the melting enthalpy of 100% crystalline PPS, which is 76.5 J/g [32], and x is the weight fraction of MWCNT.

An impact of MWCNTs on thermal diffusivity of PPS-based composites was determined using the laser flash apparatus (LFA 447, Netzsch, Selb, Germany) at a temperature range from 50 °C to 250 °C with steps of 50 °C. Measurements were divided into two groups. The thermal diffusivity of each type of sample was calculated as an average of the measurements of the four samples. Moreover, at each temperature setpoint for each sample, the measurement was repeated five times. Samples had similar dimensions with an approximate diameter d = 25 mm and height h = 1 mm.

To examine the influence of the processing temperature and the MWCNT content, the electrical resistivity of the produced materials was measured using a Keithley 6221/2182A (Cleveland, OH, USA) nano voltmeter and a DC source, equipped with copper electrodes. To ensure very good contact between the samples and the electrodes, a silver conductive paste (CW7100, Chemtronics, Kennesaw, GA, USA) was used. Five samples of each material with a diameter of 25 mm and thickness of 1 mm were tested in the range from 1 nA to 1 mA; additionally, they were cleaned with ethanol before testing. To reduce noise and thermoelectric effects, the tests were performed in the delta mode using the four-point method.

## 3. Results and Discussion

### 3.1. Rheological Properties

**Figure 1:**
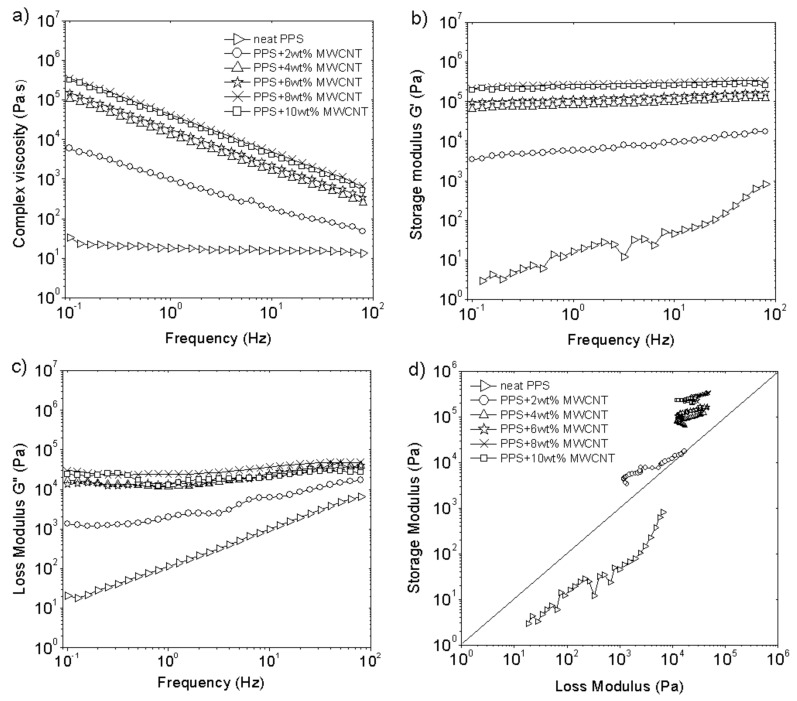
Rheological properties of PPS/MWCNT composites: (**a**) complex viscosity as a function of frequency; (**b**) storage modulus as a function of frequency; (**c**) loss modulus as a function of frequency, (**d**) Cole-Cole plot. The test was performed at 320 °C with 10% strain.

MWCNTs are known as additives that change the rheological properties of different thermoplastic and thermoset polymers to a large degree due to their high aspect ratio [33]. For the prepared PPS-based composites, the complex viscosity, storage, and loss modulus also change in the presence of MWCNTs, which is presented in Figure 1a, b, and c, respectively. Neat PPS possess a viscosity around 10^1^ Pa·s, and it is increased by about three orders of magnitude up to the value of 10^4^ Pa·s after the addition of 2 wt% MWCNTs. For the higher MWCNT loadings, the viscosity increases up to 105 Pa·s. There is no significant difference between the MWCNT concentration, especially for 8 and 10 wt%, where the curves are practically the same. The MWCNTs’ effect on the viscosity of PPS is more pronounced at low concentrations (2 wt%) and low frequencies, which has been reported in the literature for other thermoplastic polymers [34]. It should be noted that neat PPS behaves as a Newtonian liquid with no changes in the complex viscosity within the frequency range. The addition of MWCNTs causes the disappearance of a Newtonian plateau, and all PPS/MWCNT composites show a typical shear-thinning behavior with decreasing viscosity and frequency. Similar to viscosity, storage and loss modulus increase in the presence of MWCNTs. This is an effect of the increased viscosity, and strong interactions occur between the filler and the polymer. Both moduli are raised to the value of 10^3^ Pa for 2 wt% MWCNTs. Higher MWCNT loadings cause an increase in storage and loss modulus of up to 10^4^ Pa·s or 10^5^ Pa·s, respectively. Similar to the effect of viscosity, 4, 6, 8, and 10 wt% MWCNT content has a lower effect on storage and loss modulus. The character of the curves changes from a steep course to a frequency-independent plateau-like behavior. The literature has reported the same character of both moduli at high MWCNT concentrations, e.g., above 2 wt% for PEEK [35]. This is related to the rheological percolation threshold, defined as the minimum filler concentration at which the motion of the polymer chains is restricted. It can be determined from the so-called Cole-Cole plot, which is a dependence between loss and storage modulus in a logarithmic scale, as shown in Figure 1d. Neat PPS presents a linear relationship between the moduli. There is a clear divergence from the linear dependence for all composites, which means that the filler forms the structural network resulting in a more heterogeneous system. Based on the changes in storage and loss modulus, the rheological percolation threshold can be found between 0–2 wt% MWCNTs within the same range reported for PPS/MWCNT composites prepared by direct components mixing [17]. Finally, it should be noted that for neat PPS, the loss modulus (Figure 1c) exceeds the storage modulus (Figure 1b). Within the whole frequency range, neat PPS reveals more viscous behavior of the material. The situation alters after the addition of MWCNTs, and for all composites, the loss modulus is lower than the storage modulus, which results in a more elastic material.

### 3.2. Microscope Observations

**Figure 2:**
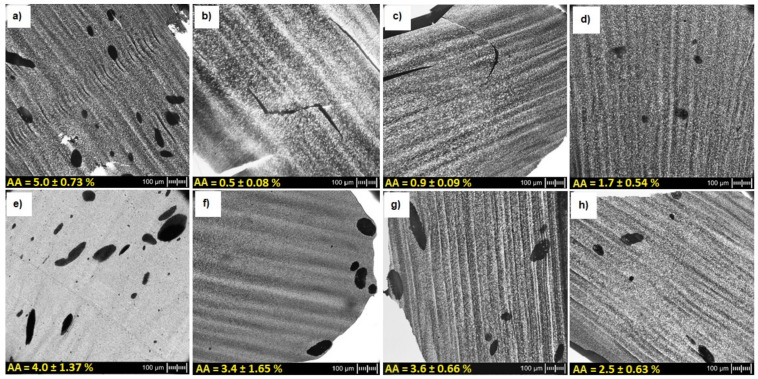
Optical micrographs illustrating the state of MWCNTs’ agglomerate dispersion at different temperature and MWCNT content: (**a**) PPS + 10 wt% MWCNT masterbatch, (**b**) PPS + 10 wt% MWCNT (T = 290 ℃), (**c**) PPS + 10 wt% MWCNT (T = 305 ℃), (**d**) PPS + 10 wt% MWCNT (T = 320 ℃), (**e**) PPS + 2 wt% MWCNT (T = 320 ℃), (**f**) PPS + 4 wt% MWCNT (T = 320 ℃), (**g**) PPS + 6 wt% MWCNT (T = 320 ℃), (**h**) PPS + 8 wt% MWCNT (T = 320 ℃).

The optical observations of non-extruded PPS + 10 wt% of MWCNTs were performed and the result is shown in Figure 2a. To investigate the MWCNTs’ agglomerate dispersion, 10 wt% MWCNT masterbatch was processed at three different temperatures: 290 °C, 305 °C, and 320 °C, and the optical microscopy observations are presented in Figure 2b–d. A slight effect of temperature on the increase in the number of agglomerates was observed. This was the effect of a decrease in viscosity with an increase in temperature. However, in each of the analyzed cases, the number of MWCNT agglomerates was at a satisfactory, low level. Moreover, the effect of MWCNT content on the agglomerates dispersion was examined for the selected temperature (320 °C), and the results are presented in Figure 2e–h. It was observed that with a lower MWCNT content, the number of agglomerates increased. This was the effect of the viscosity change, where the shear forces may be too low for lower MWCNT contents.

To evaluate the MWCNTs’ agglomerate area ratio, such optical micrographs were quantitatively evaluated by applying an image analysis procedure. The calculated CNT agglomerate area ratio confirms the decrease of agglomerates formation after applying the extrusion process to the samples and the increase in their number, along with the decrease in the content of CNTs. The influence of twin-screw extrusion conditions on nanotube dispersion is presented in the literature [36,37], which indicates that a high rotation speed and temperature are beneficial for dispersing and evenly distributing the CNT in the polymer matrix. It was found that the extrusion screw can ensure the introduction of high shear forces for CNT deagglomeration in the PPS polymer matrix. Furthermore, a better dispersion quality was achieved at higher CNT contents in the PPS matrix [38] as well as the poly(lactic acid) matrix. It was found that shear stresses as well as the viscosity of nanocomposites play an important role in destroying agglomerated nanofillers [39]. Thus, the viscosity increase resulting from the higher MWCNT content leads to higher maximum shear stresses applied to remaining CNT agglomerates. With lower CNT contents, the shear stresses can be insufficient for agglomerate breakdown.

### 3.3. Thermal Properties

The results of the thermal analysis were collected in Table 1. The thermal stability, which is understood as the temperatures at 2% and 5% weight loss, was determined from the TGA curves. The degradation starts at 453 °C (2%) and 481 °C (5%) for neat PPS. In the starting masterbatch containing 10 wt% MWCNTs, the degradation temperatures are shifted to about 12–14 °C, and the temperature of the maximum weight loss rate also increases by about 14 °C. Further processing of the masterbatch by extrusion process resulted in the values of T2% and T5%, which are about 3 °C higher for some of the composites, or lower for others. 

The temperature of the maximum weight loss is significantly improved by about 10–17 °C for all composites compared with the maximum weight loss rate. There is no apparent effect of the extrusion temperature and MWCNT content on the thermal stability of PPS/MWCNT composites. Because both T2% and T5% are the highest for the masterbatch, the additional processing step decreases the thermal stability in view of initiating the degradation process. The calculated amount of the agglomerates is presented in Figure 2. Many more MWCNT agglomerates were found in the masterbatch than in the composites after processing. It seems that the presence of agglomerates is more critical for thermal stability improvement than perfectly dispersed MWCNTs. More agglomerates in the composites hinder the flux of degradation products more effectively than single MWCNTs. It might also be that PPS chains near the MWCNT agglomerates degrade slowly, but only if they are homogenously dispersed [40]. The other possible mechanism is that the second extrusion step causes some degradation of the PPS chains, and therefore, the composites start to decompose at a lower temperature. Most of the nanofiller works as a nucleating agent for the polymers.

**Figure 3:**
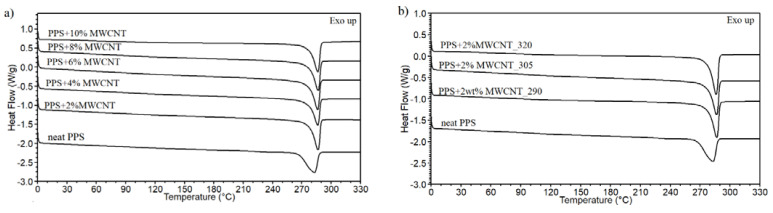
The second heating curve for the PPS/MWCNT composites. (**a**) Comparison of the MWCNT content. (**b**) Comparison of the extrusion temperature.

The analysis of the effect of MWCNT addition on the crystallization phenomenon of PPS was carried out using the DSC method. The determined meting point Tm, crystallization temperature Tc, and calculated crystallinity degree Xc are included in Table 1. The melting point of neat PPS is 283 °C, and it shifts to the value of 286–287 °C. An example thermogram comparing the effect of MWCNTs on the melting point and extrusion temperature is shown in Figure 3a,b.

**Figure 4:**
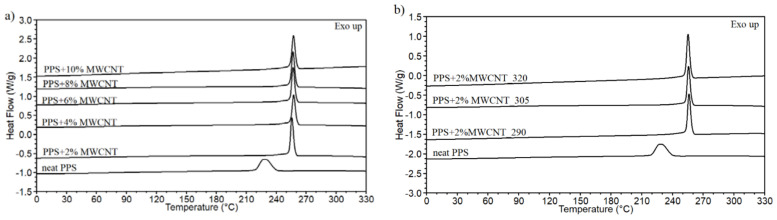
The cooling curves for the PPS/MWCNT composites. (**a**) Comparison of the MWCNT content. (**b**) Comparison of the extrusion temperature.

There is no effect of the MWCNT content and extrusion temperature on the melting point. Moreover, the melting peak of neat PPS is broad, and it is getting narrower for PPS/MWCNT composites, which reveals the changes in the crystal phase of the polymer, as demonstrated by an increase in the crystallinity degree and crystallization temperature. As shown in Table 1, for most of the composites, the addition of MWCNTs causes an increase in the crystallinity content at a maximum of 18% and an increase in the crystallization temperature of 29 °C. The nucleation effect of MWCNTs on PPS is visible by a significant shift in the crystallization peak, as presented in Figure 4a,b.

The graphs also confirm no evident influence of MWCNT concentration and extrusion temperature on the crystallization phenomena. It is confirmed by a similar value of the crystallization temperature (256–258 °C) obtained for all composites. A similar lack of linear dependence between crystallinity content and nanotube concentration was found in PA6 containing MWCNTs [41]. In the other PPS/MWCNT composites prepared by direct components mixing, the nucleating effect of MWCNTs was observed, but only for small nanotube concentrations. At higher concentrations, the melting point and crystallinity degree show a gradual decrease. Probably, the created MWCNT network hinders the movement of the polymer chains leading to forming of an imperfect crystal phase [17].

**Figure 5:**
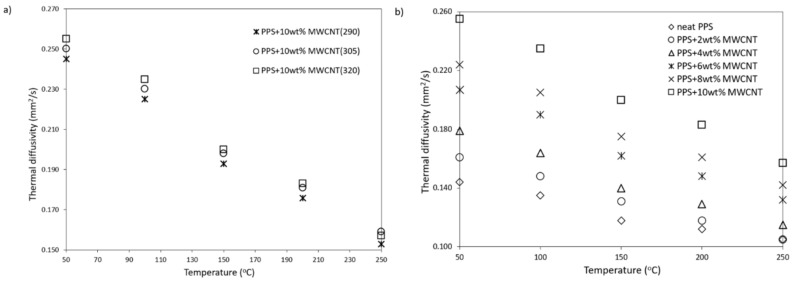
Thermal diffusivity vs. (**a**) temperature of PPS-based composites filled with MWCNT and (**b**) temperature of PPS-based composite manufactured with different temperature.

The thermal diffusivity of all PPS/MWCNT composites is shown in Figure 5a,b. Figure 5a presents the thermal diffusivity decreasing with temperature. In addition, MWCNTs affect the thermal diffusivity of PPS-based composites. The higher the weight fraction of MWCNTs, the higher the thermal diffusivity. Maximum value a = 0.255 mm^2^·s^−1^ was determined for samples PPS+10 wt% MWCNT at the temperature T = 50 °C. On the other hand, neat PPS has lower thermal diffusivity of a = 0.144 mm^2^·s^−1^ at the same temperature. That compared with samples containing 10 wt% MWCNT gives a maximum increase of approximately 77%. At maximum measured temperature T = 250 °C, the thermal diffusivity of a sample with maximum loading increases by 38% compared with neat PPS. The increase in thermal diffusivity with an increasing MWCNT weight fraction is close to linear. The achieved increase in thermal diffusivity of PPS/MWCNT composites with this type of filler is rather low when compared with other authors [19]. However, since a different filler was used, the carbon MWCNT feature is extremely high for a specific surface area. It is higher than in other allotropes of carbon-based fillers and causes higher interfacial resistance, which deteriorates heat conduction through the material.

Figure 5b shows the impact of the extrusion temperature on the thermal diffusivity of the tested samples. The higher the temperature, the higher the thermal diffusivity, but it increases the maximum by approximately 4% for samples PPS + 10 wt% MWCNT (T = 320 °C) compared with PPS + 10 wt% (T = 290 °C).

### 3.4. Electrical Properties

**Figure 6:**
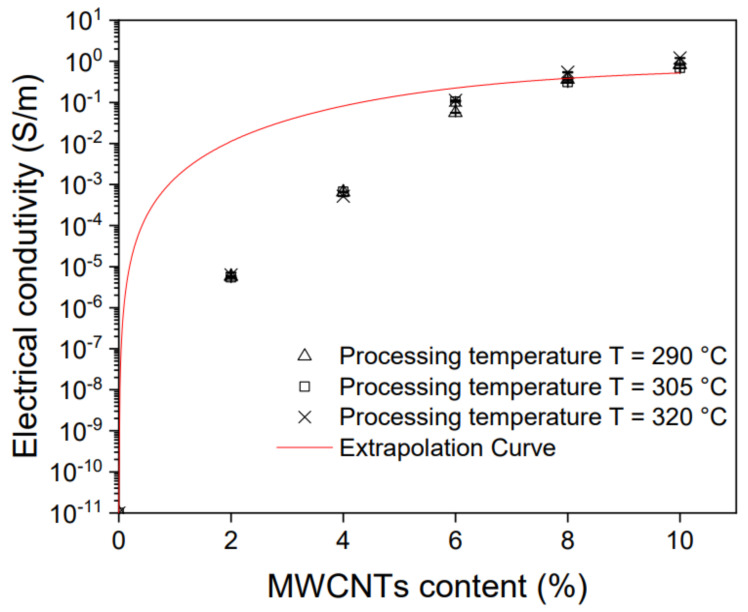
Electrical conductivity of PPS/MWCNT nanocomposites.

The influence of the MWCNT content and processing temperature on the level of electrical conductivity of PPS/MWCNT nanocomposites has been examined and the results are presented in Figure 6. The largest increase in electrical conductivity was observed from 10^−11^ S/m for neat PPS to 7·10^−5^ S/m for 2 wt% MWCNT, and this increase was almost six orders of magnitude. Then, the increase in conductivity slowed down and increased linearly from 2 to 6% in the MWCNT content, after which it slowed significantly, and the increase in concentration from 6 to 10% of MWCNT improved by one order of magnitude, finally reaching values of about 10^0^.

By analyzing the results and the conductivity curve, the electrical percolation threshold can be established between 0–2 wt% of MWCNTs. This value is relatively low compared with the values obtained in the literature at 5% of the MWCNT content [17], which proves good MWCNT dispersion in the PPS matrix. It was achieved by the selection of appropriate temperature and double extrusion on a twin-screw extruder. It was also the effect of using MWCNTs from Nanocyl, which possess very good properties as a conductive filler. Using them to make conductive nanocomposites is known in the literature and the obtained percolation threshold for these MWCNTs is about 1wt% [42,43]. Moreover, it was observed that the change of the processing temperature from 290 °C to 320 °C did not affect the electrical conductivity. The greatest change was observed for nanocomposites with 10 wt% MWCNT content, where the electrical conductivity was 8.39·10^−1^ S/m and 1.05·10^0^ S/m for 290 ℃ and 320 ℃, respectively. This is the result of a slight difference in the state of MWCNTs’ agglomerate dispersion, which is presented in Figure 2.

## 4. Conclusions

The present work investigated the effect of processing temperature and the content of carbon nanotubes on the properties of nanocomposites based on PPS. The studies aimed to select the best material and processing parameters for the production of thermoplastic veils with the addition of MWCNTs, as a unique technique to improve the properties of CFRP. For this purpose, PPS/MWCNT nanocomposites were produced at three different temperatures and with different filler contents. TGA analysis showed a slight decrease in thermal stability for the produced nanocomposites compared with a masterbatch with an MWCNT content of 10 wt%. This may be the result of the subsequent processing of the material, as the MWCNT content was not found to affect thermal stability. In the case of DSC analysis, the content of MWCNTs and the processing temperature do not affect the melting point; however, the addition of MWCNT causes an increase in the degree of crystallinity and the crystallization temperature. The highest values of thermal diffusivity and electrical conductivity were observed in nanocomposites containing 8–10 wt% MWCNTs, which was caused by the excellent thermal and electrical properties of MWCNTs. Viscosity studies showed that the addition of 2% MWCNT makes the material more elastic than plastic. Additionally, no significant effect of the amount of filler on the rheological properties was observed for higher MWCNT concentrations. In the case of microscopic observations, a larger area of agglomerates was observed for lower concentrations of MWCNTs, which is the result of lower nanocomposite viscosity, and consequently, lower shear forces during processing. The same was observed as regards the influence of the processing temperature—as the processing temperature increases, the viscosity decreases, which translates into lower shear forces and worse homogenization of the nanocomposite. Taking into account the obtained results, it was found that the content of MWCNTs within the range of 8–10 wt% and the processing temperature from 305 to 320 °C allows for obtaining the desired properties of the PPS/MWCNT nanocomposites, which will be the basis for thermoplastic nonwovens, used to improve the properties of CFRPs.

## Figures and Tables

**Table 1:** The results of the thermal analysis.

Material	Extrusion Temp. (°C)	TGA			DSC		
		T_2%_ (°C)	T_5%_ (°C)	T_d_ (°C)	T_m_ (°C)	X_c_ (%)	T_c_ (°C)
neat PPS		453	481	521	283	54.5	228
PPS + 10% masterbatch		467	493	535	286	59.3	257
PPS + 2% MWCNT	290	456	486	531	287	66.5	256
PPS + 4% MWCNT		452	484	532	287	53.2	257
PPS + 6% MWCNT		451	483	535	286	61.8	257
PPS + 8% MWCNT		453	484	533	286	58.7	257
PPS + 10% MWCNT		451	482	530	287	65.3	257
PPS + 2% MWCNT	305	454	487	535	287	57.3	256
PPS + 4% MWCNT		453	484	531	286	53.3	258
PPS + 6% MWCNT		454	485	534	287	58.9	257
PPS + 8% MWCNT		454	484	534	286	73.1	257
PPS + 10% MWCNT		457	486	535	286	61.1	257
PPS + 2% MWCNT	320	452	484	533	286	60.6	255
PPS + 4% MWCNT		457	487	532	287	67.5	257
PPS + 6% MWCNT		454	484	534	287	65.7	257
PPS + 8% MWCNT		456	486	538	287	64.1	257
PPS + 10% MWCNT		453	483	534	286	54.1	257

## Data Availability

All data are available in the main text.
